# Round the Hospitals

**Published:** 1920-11-06

**Authors:** 


### ROUND THE HOSPITALS.

The following ladies were personally decorated by H.M. the King at an Investiture held in the Ballroom of Buckingham Palace on November 2:—Order of the British Empire (Military Division) (Officer).—Queen Alexandra's Imperial Military Nursing Service Reserve: Miss Nora Easby, also received Royal Red Cross (First Class). Bar to the Royal Red Cross.—Territorial Force Nursing Service: Misses Henrietta Hannath and Mary Laing. Royal Red Cross and Bar.—Territorial Force Nursing Service: Miss Edith MacFarlane. Royal Red Cross (First Class).—Queen Alexandra's Imperial Military Nursing Service: Misses Eva Ellis, Vaughan Forrest, Clara Johnson, Dinah Macgregor. Reserve: Misses Elizabeth Crawford, Katherine Fitton, Annie Hulbert, Winifred Ramsey, Edith Sparks. Territorial Force Nursing Service: Misses Dora Chapman, Helen Fergusson, Winifred Friend, Sophia Hatton, Minnie Holmes, Annie Howard, Mabel Jennings, Edith Kilburn, Mary Langlands, Beatrice Sanderson. Civil Nursing Service: Miss Mary Sinzininex. British Red Cross Society: Miss Edith Sheriff-MacGregor.

---

A meeting of the General Nursing Council will be held at 2 p.m. on Friday, November 12, 1920, in Room III, third floor, Ministry of Health, Whitehall.

---

The long-expected Army Order relating to matrons' pay is now issued. It states that the minimum rate of pay of a matron-in-chief of the Q.A.I.M.N.S. will be £425, rising by increments of £15 to £470, while the pay of a principal matron will be £260, rising by increments of £12 to £296. These rates will have effect from April 1 last. Retired pay will be assessed on the scale of £3 for each year of service, with the addition of a rank element as follows: Matron-in-chief after one year as such, £170, with £40 for each further year up to a maximum of £290; principal matron, after one year as such, £100, rising by £12 a year to £148. The maximum rate of retired pay in both service element and rank element will be £370 for matron-in-chief and £220 for principal matron. It is also provided that board and washing allowance for members of the Q.A.I.M.N.S. and the permanent nursing establishment of military families' hospitals will be 3s 6d. a day at home and abroad. When free messing or hospital diets and extras are provided the board and washing allowance is reduced to 7d. a day.

---

It seems we cannot get on without the V.A.D.s. The War Office announces it must continue the employment of certain officials and members of the V.A.D. General Service Section after October 31, and they are asking them to make a fresh engagement to serve till April 30, 1921, or as long as they are needed, whichever is the shorter period. Rates of pay remain as at present. Some who are serving with the Black Sea Army (how many people were aware that we possessed a Black Sea Army?) will be given an allowance of 3s. a day as from November 1.

---

It appeared at one time as if there might be a delay in the proposal of the Bermondsey Board of Guardians to enlarge the nurses' home attached to their infirmary. The plans were submitted to the Ministry of Health, and on October 16 their sanction was formally sent to the guardians. The extension will cost £38,450, and will provide additional accommodation for sixty-two nurses, with mess-room and kitchen. A letter was then received from the Architect's Department of the London County Council stating that they are considering this building under the Housing (Additional Powers) Act, which empower the council to prohibit these works. "Pending the decision of the council in this case," the letter concluded, "any work which may be proceeded with will be at your own risk." There was also a suggestion that the guardians should give an undertaking to eliminate all brickwork and substitute some form of concrete construction. Such an alteration in the specifications would have meant considerable delay, and probably there might have had to be fresh tenders. Happily, however, pressure was brought to bear on the officials at Spring Gardens, with the result that all opposition was withdrawn, and the work already commenced.

---

Objections have been made, it appears, by certain members of the Visiting Committee of the Swansea Poor-Law Infirmary to the practice of probationer nurses possessing less than half a year's service being given night duty. The matter was considered by the Committee at its meeting last week, when, after, discussion, a sub-committee was instructed to survey the position and report. The superintendent nurse put forward the necessity of placing probationers on night duty when there is a limited staff, and her approval of the system is reinforced by Dr. Edwards, who told the Committee that this has always been done in institutions and hospitals. There are many institutions besides that at Swansea where the younger probationers are given night duty, and it would be instructive to learn from them whether the system has given rise to complaints, either from the probationers themselves, from the superintendent nurse, or the night sisters in charge. On the face of it, it is not an ideal arrangement.

---

Swansea Corporation Joint Staff Committee proposes that the scale of salaries of school nurses and health visitors be increased to a minimum of £170, rising to £245 by six annual increments of £12 10s.; that each person in the old scale of £156, rising to £208, be placed in the new scale in their relative position as from January 1, 1920, with an increment of £12 10s., from April 1, 1920. This is quite good.

Recently the Camberwell Board of Guardians appointed a sub-committee to consider the question as to the feeding and care of the nurses. Now the sub-committee expresses the opinion that it would be greatly to the advantage of the infirmary if the matron were relieved of the household duties in connection with the nurses' dietary and a skilled housekeeper appointed to carry out this work. This would enable the matron to be free for her administrative work. The committee suggests that the housekeeper should be paid £100 a year, with emoluments.

---

It is most gratifying to learn that the hours of the nursing staff at the Royal Infirmary, Sheffield, are to be reduced. This has been rendered possible by a grant of £5,000 from the British Red Cross Society, which has enabled the Board to add another storey, containing twenty-one bedrooms and sanitary blocks, to the existing Nurses' House. The nursing staff, as will have been noticed from our advertisement columns, is being increased, and applications from probationer nurses are invited. There has also been added to the nurses' quarters a new recreation-room, with a special floor for dancing. It is proposed in the near future to appoint a new sister to take over part of the duties now performed by the assistant matron and home sister. This new sister will be home sister and sister-tutor, and by this means it is hoped to keep the infirmary right up to date and in the forefront of the English nurse-training schools.

---

A pleasant little ceremony took place at the meeting of the City of London Guardians at 61 Bartholomew Close recently when Miss Marian Marsh-MacNeill, who has been appointed nurse-midwife by the Thavies Inn Committee, received from the Chairman the decoration of the Royal Red Cross, Second Class, awarded to her after four years' service as sister in the Territorial Force Nursing Service. Sister MacNeill was trained at the City of London Infirmary, and much gratification was expressed at the distinction she had won. Short of receiving the decoration from the hands of the King, perhaps no better way of marking the honour can be suggested than this formal presentation by the Board under which the recipient is serving.

---

Considerable commotion has been caused in Dundee by the decision of the Fife doctors, through the County Branch of the British Medical Association, to impose a minimum tariff of one guinea per lecture upon all V.A.D. Corps requiring the services of a practitioner as lecturer. It is feared that this decision will entirely put a stop to the development of Red Cross work in the district. It was inevitable, however, that the generous response made by doctors to demands for the free instruction of amateur nurses should cease after the war. If it is for the public good that such classes should be held, the public and the members of the classes should defray the cost of lecturers. We would go farther. We think that the nursing sisters who have so freely and generously helped to build up the army of Red Cross workers are entitled to remuneration for time expended in giving bandaging classes and other lessons. The pupils are often far better able to afford to pay fees than the doctors and nurses to give time.

---

The sum of £250 was realised by the sale held at York in the Treasurer's House in aid of the York District Nursing Fund. This annual event is always well supported in the city. One of the interesting features this year was the baby clothing on the nurses' stall, which was provided by the present staff of nurses at the Purey-Cust Nursing Home.

---

The Newcastle City Council have referred back by one vote a report presented by the Sanitary Committee proposing an increase of salary for the health visitors and tuberculosis visiting nurses, based on the Civil Service scale already in operation in respect to other Corporation employees. The proposed increases amounted to £1,841 per annum, half of which would be paid by the Ministry of Health. One of the opposing Councillors contemptuously asserted that "the very best nurses in the city were only getting £60 a year and all found." The matter has been promptly taken up by the members of the Northumberland and Durham Centre of the College of Nursing, who, after a meeting at their club in the Windsor Terrace, Newcastle, sent a protest to the Council. They directly controvert the statement that £60 is the high-water mark of nurses' salaries in the city, and urge that economy should be shown, not by paying public health nurses an inadequate salary, but by introducing better organisation. To employ fewer nurses and entrust them with the whole of the duties in their district, instead of setting several nurses to visit the same house in a different capacity, would obviate much waste of time and money. It is a great advantage for the nursing profession to have a body able to speak with authority and knowledge in matters of this description, and we cordially agree with our correspondent, "I. M.," that the affair contains "an obvious lesson to fully trained nurses." It is to join the College, and thus strengthen its hands.

---

We learn that Miss Elsie Catherine Pape, who has trained at King's College Hospital and has for the past year been sister of the radiological department, has resigned to take up an important appointment in the radiological section of a large hospital at Shanghai. She will be much missed at her *alma mater*. Miss Elsie S. Cook, who was trained at King's, and has held the appointment of night sister for the past year, has been appointed home sister in place of Miss Grace Wilson, who to the great regret of all, has recently resigned this office. The new sister of the radiological department is Miss Annie Slater, who was trained at King's, and has recently held the post of sister of the *x*-ray department at the Norfolk and Norwich Hospital.

